# Clinical Efficacy of Routinely Administered Belimumab on Proteinuria and Neuropsychiatric Lupus

**DOI:** 10.3389/fmed.2020.00222

**Published:** 2020-05-27

**Authors:** Marlene Plüß, Björn Tampe, Noah Niebusch, Michael Zeisberg, Gerhard A. Müller, Peter Korsten

**Affiliations:** Department of Nephrology and Rheumatology, University Medical Center Göttingen, Göttingen, Germany

**Keywords:** systemic lupus erythematosus, lupus nephritis, neuropsychiatric lupus erythematosus, belimumab, monoclonal antibodies

## Abstract

**Background and Objectives:** Belimumab (BEL) is a monoclonal antibody approved for the treatment of active systemic lupus erythematosus (SLE) but not for lupus nephritis (LN) and neuropsychiatric systemic lupus erythematosus (NPSLE). We aimed to assess BEL's effects on these severe, potentially life-threatening manifestations.

**Methods:** Retrospective observational cohort study using routine clinical data in a case series of patients with SLE receiving BEL.

**Results:** Sixteen patients received BEL therapy for active SLE. Nine were excluded because they had no LN or NPSLE. Six suffered from LN, and one patient had NPSLE. All LN patients received BEL in addition to standard therapy including glucocorticoids, hydroxychloroquine, and mycophenolate mofetil in five cases, and tacrolimus in one case. Three patients with proteinuria >1,000 mg/g creatinine responded well (one complete, two partial renal responses); all other patients had decreasing proteinuria and a reduction in anti-dsDNA levels. The patient with NPSLE who had failed previous therapies had persistent clinical improvement of cutaneous and neuropsychiatric manifestations. There was one mild allergic reaction and one lower respiratory tract infection, but no other adverse events. One patient discontinued therapy due to a lack of improvement in clinical symptoms, another because of clinical remission.

**Conclusions:** In our series, BEL led to a decrease of proteinuria in patients with proteinuria of more than 1,000 mg/g creatinine despite standard of care treatment, and led to a marked clinical improvement in one patient with NPSLE. No adverse events were observed. Routinely administered BEL shows clinical efficacy on non-approved manifestations, but careful patient selection is warranted.

## Introduction

Systemic lupus erythematosus (SLE) is a rare autoimmune disease that can potentially affect every organ system (1). Many organ manifestations can be managed effectively with the available immunosuppressive therapies. Lupus nephritis (LN) and neuropsychiatric lupus erythematosus (NPSLE), however, are two of the organ manifestations that may lead to a worse overall prognosis (2, 3). LN occurs in up to 38–60% of SLE patients throughout the disease course (4, 5). The prevalence of NPSLE is more challenging to assess due to its clinical heterogeneity. A recent meta-analysis reported neuropsychiatric syndromes to be present in about 50% of all patients (6).

Belimumab (BEL) is a monoclonal antibody targeting B-lymphocyte stimulator (BLyS), and is FDA-approved as an additional treatment for SLE patients with persisting disease activity despite standard of care (SoC). It was investigated in the BLISS-52 and−76 trials (7, 8) and subsequently approved internationally; however, as LN and NPSLE were exclusion criteria in these trials, affected patients were only assessed indirectly (9). It is, therefore, as yet unclear whether or not BEL is of benefit for SLE patients with LN or NPSLE. Here, we aim to assess BEL's efficacy on LN and NPSLE patients who received BEL as in label-treatment, and report our experience using BEL in these off-label manifestations.

## Methods

### Population and Setting

This is a retrospective observational single-center cohort study of a case series of SLE patients treated at our center who received BEL in addition to SoC. BEL was initiated for continued SLE disease activity (clinical and/or serological) despite SoC medications. Continued disease activity was defined as frequent flares and requirement for repetitive increases of prednisolone doses, or the inability to taper down prednisone to a dose below 7.5 mg daily. Patients who had flares mostly suffered from relapsing arthritis or persistent skin disease with lower prednisone doses. We collected routine clinical and laboratory data and report on the relevant outcomes for the off-label manifestations, LN, and NPSLE, respectively. Of note, BEL was not specifically initiated for LN or NPSLE, and all patients with LN underwent SoC medication including cyclophosphamide (CYC) or mycophenolate mofetil (MMF). All patients were followed consecutively at regular intervals.

### Data Selection and Recording

The data used for analysis consisted of routinely collected clinical and laboratory data. Patients who regularly attend our outpatient clinic were screened for eligibility. Routine clinical and laboratory assessment included the parameters described in the SLE disease activity index 2000 (10); in addition, complete blood count, serum creatinine, blood urea nitrogen, erythrocyte sedimentation rate, c-reactive protein, urinalysis for proteinuria [reported as mg/g creatinine from spot urine as an estimation of proteinuria over 24 h (mg/d)] as well as anti-double-stranded (ds) DNA antibodies and complement factors C3 and C4. Renal responses were defined as complete (<650 mg/d after 6 months), partial (reduction, but >650 mg/d after 6 months), and no response (11).

### Statistical Methods

Descriptive statistics were used to characterize the study population. Before-and-after comparisons were used for the assessment of therapeutic effects. Comparisons between groups were assessed using Friedman's test or Kruskal-Wallis Test. All statistical tests were performed using GraphPad Prism (Version 8.2.1 for mac OS, GraphPad Software, La Jolla California USA, www.graphpad.com).

### Ethics Statement

While no formal approval is required for the use of routine clinical data, the study protocol was acknowledged by the local Ethics committee (No. 4/8/19), and all patients consented to the use of their routinely collected data as part of their regular medical care.

## Results

Of a total number of 16 patients with SLE treated with BEL at our center, nine were excluded due to absent LN or NPSLE. Of the remaining seven patients, six had biopsy-proven LN, and one had a clinically definite diagnosis of NPSLE.

### Effect on Lupus Nephritis

The six patients with LN receiving BEL were all female and between 27 and 52 years of age. All had biopsy-proven LN class III, IV, or V, and a positive antibody status for anti-nuclear antibodies (ANA) and anti-ds DNA antibodies (Table 1). All patients received BEL intravenously and were followed up at regular intervals. Data collected three and six months after the initiation of BEL therapy are presented in Table 1.

**Table 1:** Clinical and laboratory characteristics of the study population.

**Patient**	**Age, sex, ethnicity**	**Disease duration (years)**	**Antibodies, complement levels**	**Renal biopsy (time before BEL initiation)**	**LN class**	**Previous therapies**	**Proteinuria (mg/g creatinine) (Baseline, after 6 months [% reduction])**	**Current therapy**	**GC (mg/d) (Baseline, after 6 months [% reduction])**
1	49, f, Asian	15	ANA+, dsDNA+, SSA+, SSB+, Histone +, C3↓, C4↓	1 month	IV–G	GC, CYC, HCQ, MMF	4,074, 1202 (−70.5%)	GC, MMF	50, 2.5 (−95%)
2	52, f, Caucasian	19	ANA+, dsDNA+	12.5 years	V–IV	GC, HCQ, TAC	117, 76 (−35.04%)	GC, HCQ, TAC	5, 5 (0%)
3	30, f, Caucasian	4	ANA+, dsDNA+, SSA+, SSB+, Histone +, C3↓, C4↓	4 months	IV–G (A)	GC, HCQ, MMF	346, 162 (−53.18%)	GC, HCQ, MMF	60, 4 (−93.3%)
4	27, f, Caucasian	8	ANA+, dsDNA+, C3↓	13 months	III (A, C)	GC, HCQ, MMF	489, 115 (−76.48%)	GC, HCQ, MMF	6.5, 5 (−15.3%)
5	35, f, Caucasian	4	ANA+, dsDNA+, Histone +, C3↓, C4↓	4 months	III A	GC, MMF	159, 74 (−53.46%)	GC, AZA, HCQ	15, 5 (−66.3%)
6	40, f, Caucasian	17	ANA+, dsDNA+, APLA+	2 months	IV	GC, CYC, AZA, MMF	4,420, 121 (−97.26%)	GC, MMF	15, 5(−66.6%)
7	75, m, Caucasian	19	ANA+, dsDNA+, U1snRNP+, Histone+, C3↓, C4↓	17 years	None	GC, CYC, AZA, MTX, RTX	1,783, 655 (−63.26%)	GC, AZA	20, 2.5 (−87.5%)

*ANA, antinuclear antibody; APLA, anti-phospholipid antibodies; AZA, azathioprine; C3/4, complement factor 3/4; CYC, cyclophosphamide; dsDNA; double-stranded DNA; f, female; GC, glucocorticoids; HCQ, hydroxychloroquine; LN, lupus nephritis; m, male; MMF, mycophenolate mofetil; MTX, methotrexate; RTX, rituximab; TAC, tacrolimus*.

### Case #1

This 49-year-old patient of Asian ancestry had severe proteinuria of more than 4,000 mg/g creatinine and received BEL in addition to treatment with glucocorticoids (GC), hydroxychloroquine (HCQ), and mycophenolate mofetil (MMF) starting 1 month after renal biopsy. She developed allergic symptoms (fever, erythematous rash), and BEL was discontinued; upon re-exposition, however, no further allergic symptoms have developed, and the patient continues BEL therapy. Proteinuria was markedly reduced during BEL treatment but remained >650 mg/g creatinine after 6 months. She had a partial renal response.

### Case #2

This 52-year-old Caucasian patient had undergone a renal transplant for LN almost 13 years before the initiation of BEL, and developed transplant kidney LN. Her background therapy consisted of GC, HCQ, leflunomide (LEF), and tacrolimus (TAC), the latter as part of her post-transplant immunosuppression. With BEL, the patient had mild improvement of proteinuria and clinically stable disease, but no additional benefit. Therefore, BEL and LEF were discontinued after five months as per the patient's wishes. It has to be noted, however, that the patient did not have significant proteinuria at the initiation of BEL.

### Case #3

This further Caucasian patient, 30 years of age, received BEL in addition to GC/MMF/HCQ for relapsing arthritis and malar rash four months after her renal biopsy. A further reduction by about 50% after six months of treatment could be demonstrated (Table 1). She was also able to taper down her steroid dosage and experienced overall improvement of her quality of life.

### Case #4

This 27-year-old patient received BEL in addition to GC, HCQ and MMF starting 13 months after renal biopsy and was shown to have a significant albeit less pronounced reduction in proteinuria (−35%, Table 1). She experienced a clinical remission of SLE and wished to discontinue the additional treatment later on.

### Case #5

This 35-year-old Caucasian patient received BEL in addition to GC/MMF for persisting skin disease and arthritis starting four months after renal biopsy. She exhibited another significant reduction in proteinuria of 53% after six months. She was also able to reduce her GC doses and reported an overall improvement of quality of life.

### Case #6

This patient had undergone multiple treatment regimens, including azathioprine (AZA) and cyclophosphamide (CYC) for severe LN class IV with high-grade proteinuria and stage 4 chronic kidney disease. She had experienced multiple infectious complications requiring antibiotic therapy. Her background regimen consisted of GC and MMF when BEL was initiated two months after her most recent renal biopsy. She had a significant reduction in proteinuria from >4,000 mg/g creatinine to just over 100 mg/g creatinine. In light of further respiratory and urinary tract infections, the dose was reduced, and the administration of BEL was switched from intravenous to subcutaneous application. The patient continued to benefit from BEL therapy and has remained on the treatment for over two years.

### Case #7—Effect on Neuropsychiatric Lupus

The patient with NPSLE is a Caucasian gentleman with a diagnosis of SLE at the age of 56 and presented with multi-organ involvement including skin erythema and ulcerations, arthralgia, myalgia, bicytopenia, and pulmonary fibrosis with subsequent pulmonary arterial hypertension. During a flare in 2016, he had developed cerebral manifestations with dysarthria, severe immobilizing ataxia, and concentration deficits. The patient failed or could not tolerate multiple therapies, including HCQ, CYC, MMF, LEF, and AZA, as well as rituximab (RTX) so that the cutaneous and neuropsychiatric disease manifestations remained insufficiently controlled. Therefore, we decided to initiate treatment with BEL, which was given in conjunction with three doses of methylprednisone (250 mg) and rapidly tapered down. After three intravenous infusions with BEL the cutaneous and, most importantly, neuropsychiatric symptoms (dysarthria and ataxia) improved markedly, enabling the patient to continue a largely independent lifestyle with minimal assistance. Also, findings on cerebral magnetic resonance imaging (MRI) stabilized over time (Figure 2). Additionally, this patient also had proteinuria, but he did not have biopsy-proven LN. His first renal biopsy was performed 17 years before BEL initiation. Later on, we performed another renal biopsy in this patient due to increasing proteinuria, which showed focal segmental glomeruosclerosis unrelated to SLE.

**Figure 1:**
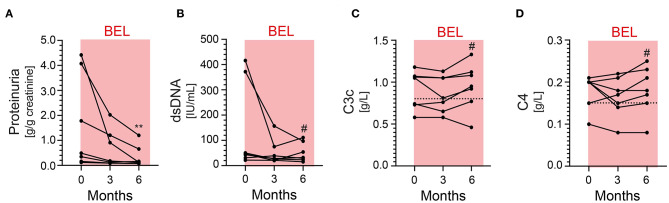
Proteinuria, anti-double-stranded (ds) DNA antibodies and complement levels C3 and C4 at baseline and up to 6 months in patients with LN receiving BEL in addition to standard therapy. **(A)** There is a statistically significant reduction of proteinuria after 6 months (**p = 0.0015). The net effect is most pronounced in patients with the highest degree of proteinuria. **(B)** anti-dsDNA antibodies over time. A non-statistically significant decline is observed in patients with the highest baseline levels. **(C,D)** Complement factors C3 and C4 over time. There is no statistically significant difference after 6 months. Dotted lines represent the lower limit of normal (normal range of C3 0.82–1.93 g/L, C4 0.15–0.57 g/L). BEL, belimumab; LN, lupus nephritis. ^#^not significant.

**Figure 2:**
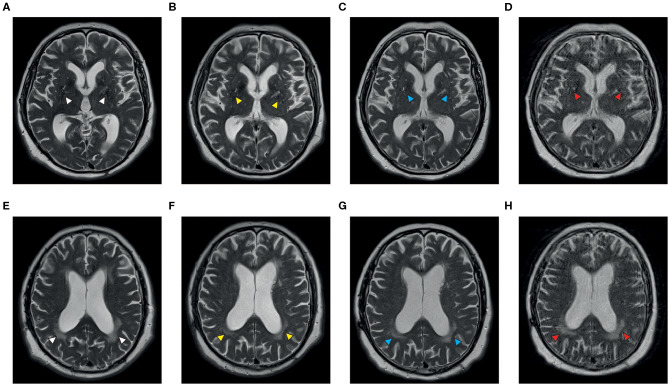
Axial T2-weighted MR images of a patient with NPSLE before **(A,B,E,F)** and during treatment with belimumab **(C,D,G,H)**. Vascular white matter lesions can be demonstrated in the region of the basal ganglia **(A)** and the posterior periventricular matter **(E)** at the onset of neuropsychological symptoms (white arrowheads); there is a progress of the central lesions and formation of anterior white matter involvement within 15 months while the posterior lesions appeared less intense (**B,F**, yellow arrowheads). The panels on the right demonstrate stable disease after four (**C,G**, blue arrowheads) and 22 months (**D,H**, red arrowheads) of treatment with belimumab, respectively. Images courtesy of Prof. Dr. C. Riedel, Department of Neuroradiology, University Medical Center Göttingen.

## Discussion

Of the seven patients analyzed in this study, all showed significantly reduced levels of proteinuria during treatment with BEL, ranging from −35 to −97%. The levels of anti-dsDNA antibodies remained stable or diminished further with SoC plus BEL. Complement levels showed a tendency to normalize. Perhaps most importantly, all patients were able to significantly reduce the glucocorticoid doses and reported a symptomatic improvement of quality of life, although we did not formally assess this with questionnaires. In a recent analysis of the MAINTAIN nephritis trial, cut-off values below 650 mg/d after 6 months and 700 mg/d after 12 months were associated with a more favorable renal prognosis (11). Therefore, it should be highlighted that four out of the seven patients analyzed had a proteinuria of <500 mg/g creatinine, which would be considered as complete renal remission of LN, and, therefore, not prompt an escalation of immunosuppressive therapy. We did, however, include these patients in our analysis as they had biopsy-proven LN and were given BEL for continued disease activity, which might affect their renal outcome in the long run. Another limitation of the study might be that we routinely measure proteinuria in spot urine samples in an outpatient setting. This has been shown to be a reliable alternative (12). In a meta-analysis comparing spot urine to 24-h urine collection, it was argued that both tests correlated, at best, moderately (13). These findings are limited by the fact that only three of the 13 analyzed studies used Bland-Altman analysis as appropriate test for agreement between these two different methods.

The patient with NPSLE experienced a remarkable clinical improvement that allowed him to continue a mostly independent lifestyle. It has to be noted that imaging findings do not always correlate with clinical findings in NPSLE, as MRI lesions may be found in asymptomatic patients (14).

Adjunct therapy with BEL in patients with LN or NPSLE remains off-label: According to the EULAR 2019 standard of care recommendations for LN (15), BEL should currently be considered in patients with *extrarenal* lupus manifestations with poor or no response to first-line treatment.

While the results of further prospective clinical trials are eagerly awaited, a *post-hoc* analysis of the available BLISS trial data of patients with renal manifestations at baseline concluded in favor of BEL (16). Broadening this retrospective data pool to include patients with LN beyond the BLISS trials led to the conclusion that 55.1% of LN patients showed an improvement in renal parameters with BEL, including a reduction in proteinuria of 38% (9).

Interestingly, multiple case reports have been published suggesting a benefit of BEL in patients with LN, both as an adjunct treatment when first- and/or second-line therapies have failed (17–19), and as maintenance therapy after RTX (20–22). A case in which LN was refractory to RTX and then successfully treated with BEL has also been reported (23).

On the other hand, caution has been raised by some authors who witnessed patients developing LN while undergoing therapy with BEL (24, 25). However, a recent extensive review (26) of the treatment of refractory LN concludes that BEL—in combination with RTX, and possibly as monotherapy—will play a role. In the meantime, Glaxo Smith Kline, the manufacturer of Belimumab, has released a news outline that the phase 3 study of BEL in LN reached its primary endpoint (27). The final results have not been published as of yet but are awaited eagerly.

As far as NPSLE itself is concerned, data and case reports are not available. BLyS has, however, been shown to be elevated in the cerebrospinal fluid of NPSLE patients, making it a feasible target (28). A concise review of the available evidence (29) concluded that while, again, the BLISS trials were neither designed nor powered for NPSLE research, BEL appears to have been beneficial for neuropsychiatric symptoms.

In conclusion, the data of our retrospective series of seven cases presented in this study show a favorable effect of BEL on proteinuria in patients with biopsy-proven lupus nephritis as well as on neuropsychiatric manifestations in one patient with severe NPSLE. While further randomized controlled trials and subsequent recommendations concerning these indications are awaited, this data may help other clinicians to find appropriate and safe treatment for their patients.

## Data Availability Statement

The datasets generated for this study are available on request to the corresponding author.

## Ethics Statement

The studies involving human participants were reviewed and approved by Ethics committee of the University Medical Center Göttingen. The patients/participants provided their written informed consent to participate in this study. Written informed consent was obtained from the individual(s) for the publication of any potentially identifiable images or data included in this article.

## Author Contributions

MP treated patients, collected and analyzed data, created figures, and co-wrote the manuscript. BT analyzed data, created figures, and reviewed the manuscript. NN collected data and reviewed the manuscript critically. MZ analyzed data and reviewed the manuscript. GM treated patients, analyzed data, and reviewed the manuscript. PK treated patients, conceived the study, collected and analyzed data, created figures, and co-wrote the manuscript.

## Conflict of Interest

PK has received honoraria or speaker fees (<10,000 Euros combined) from Abbvie, Bristol Myers Squibb, Glaxo Smith Kline, Janssen, Lilly, Pfizer, and Sanofi-Aventis all unrelated to this study. The remaining authors declare that the research was conducted in the absence of any commercial or financial relationships that could be construed as a potential conflict of interest.

## References

[1] Sciascia S Radin M Roccatello D Sanna G Bertolaccini ML. Recent advances in the management of systemic lupus erythematosus. F1000Res. (2018) 7:F1000. 10.12688/f1000research.13941.1 30026918 PMC6039948

[2] Faurschou M Starklint H Halberg P Jacobsen S. Prognostic factors in lupus nephritis: diagnostic and therapeutic delay increases the risk of terminal renal failure. J Rheumatol. (2006) 33:1563–9. 16881113

[3] Hanly JG Urowitz MB Su L Bae SC Gordon C Wallace DJ. Prospective analysis of neuropsychiatric events in an international disease inception cohort of patients with systemic lupus erythematosus. Ann Rheum Dis. (2010) 69:529–35. 10.1136/ard.2008.106351 19359262 PMC2929162

[4] Hanly JG O'Keeffe AG Su L Urowitz MB Romero-Diaz J Gordon C. The frequency and outcome of lupus nephritis: results from an international inception cohort study. Rheumatology. (2016) 55:252–62. 10.1093/rheumatology/kev311 26342222 PMC4939728

[5] Ward MM. Changes in the incidence of end-stage renal disease due to lupus nephritis, 1982-1995. Arch Intern Med. (2000) 160:3136–40. 10.1001/archinte.160.20.3136 11074743

[6] Unterman A Nolte JES Boaz M Abady M Shoenfeld Y Zandman-Goddard G. Neuropsychiatric syndromes in systemic lupus erythematosus: a meta-analysis. Semin Arthritis Rheum. (2011) 41:1–11. 10.1016/j.semarthrit.2010.08.001 20965549

[7] Navarra SV Guzmán RM Gallacher AE Hall S Levy RA Jimenez RE. Efficacy and safety of belimumab in patients with active systemic lupus erythematosus: a randomised, placebo-controlled, phase 3 trial. Lancet. (2011) 377:721–31. 10.1016/S0140-6736(10)61354-2 21296403

[8] Furie R Petri M Zamani O Cervera R Wallace DJ Tegzová D. A phase III, randomized, placebo-controlled study of belimumab, a monoclonal antibody that inhibits B lymphocyte stimulator, in patients with systemic lupus erythematosus. Arthritis Rheum. (2011) 63:3918–30. 10.1002/art.30613 22127708 PMC5007058

[9] Sciascia S Radin M Yazdany J Levy RA Roccatello D Dall'Era M. Efficacy of belimumab on renal outcomes in patients with systemic lupus erythematosus: a systematic review. Autoimmun Rev. (2017) 16:287–93. 10.1016/j.autrev.2017.01.010 28147262

[10] Gladman DD Ibañez D Urowitz MB. Systemic lupus erythematosus disease activity index 2000. J Rheumatol. (2002) 29:288–91. 11838846

[11] Tamirou F Lauwerys BR Dall'Era M Mackay M Rovin B Cervera R. A proteinuria cut-off level of 0.7 g/day after 12 months of treatment best predicts long-term renal outcome in lupus nephritis: data from the MAINTAIN nephritis trial. Lupus Sci Med. (2015) 2:e000123. 10.1136/lupus-2015-000123 26629352 PMC4654096

[12] Choi IA Park JK Lee EY Song YW Lee EB. Random spot urine protein to creatinine ratio is a reliable measure of proteinuria in lupus nephritis in Koreans. Clin Exp Rheumatol. (2013) 31:584–8. 23711154

[13] Medina-Rosas J Yap KS Anderson M Su J Touma Z. Utility of urinary protein-creatinine ratio and protein content in a 24-hour urine collection in systemic lupus erythematosus: a systematic review and meta-analysis. Arthritis Care Res. (2016) 68:1310–9. 10.1002/acr.22828 26714024

[14] Postal M Lapa AT Reis F Rittner L Appenzeller S. Magnetic resonance imaging in neuropsychiatric systemic lupus erythematosus: current state of the art and novel approaches. Lupus. (2017) 26:517–21. 10.1177/0961203317691373 28394232

[15] Fanouriakis A Kostopoulou M Alunno A Aringer M Bajema I Boletis JN. 2019 update of the EULAR recommendations for the management of systemic lupus erythematosus. Ann Rheum Dis. (2019) 78:736–45. 10.1136/annrheumdis-2019-215089 30926722

[16] Dooley MA Houssiau F Aranow C D'Cruz DP Askanase A Roth DA. Effect of belimumab treatment on renal outcomes: results from the phase 3 belimumab clinical trials in patients with SLE. Lupus. (2013) 22:63–72. 10.1177/0961203312465781 23263865

[17] Fließer EE Korsten P Koziolek MJ Niewold TB Patschan D Müller GA. Successful treatment of a mycophenolate mofetil-refractory proliferative lupus nephritis with belimumab in a 19-year-old woman. Lupus. (2013) 22:1523–5. 10.1177/0961203313504145 24014569

[18] Fontana F Alfano G Leonelli M Cerami C Ligabue G Spinella A. Efficacy of belimumab for active lupus nephritis in a young hispanic woman intolerant to standard treatment: a case report. BMC Nephrol. (2018) 19:276. 10.1186/s12882-018-1066-3 30342482 PMC6196012

[19] Margiotta DPE Basta F Batani V Afeltra A. Belimumab and low-doses of mycophenolate mofetil as induction therapy of class IV lupus nephritis: case series and literature review. BMC Nephrol. (2018) 19:54. 10.1186/s12882-018-0847-z 29514612 PMC5842533

[20] Kraaij T Kamerling SWA de Rooij ENM van Daele PLA Bredewold OW Bakker JA. The NET-effect of combining rituximab with belimumab in severe systemic lupus erythematosus. J Autoimmun. (2018) 91:45–54. 10.1016/j.jaut.2018.03.003 29636274

[21] Kraaij T Huizinga TWJ Rabelink TJ Teng YKO. Belimumab after rituximab as maintenance therapy in lupus nephritis. Rheumatology. (2014) 53:2122–4. 10.1093/rheumatology/keu369 25205827

[22] Simonetta F Posa M Villard J Marceau-Renaut A Preudhomme C Samii K. Restoration of hematopoiesis in a case of myelodysplastic syndrome associated with systemic lupus erythematosus treated with rituximab. Ann Hematol. (2015) 94:1247–9. 10.1007/s00277-015-2363-6 25847363

[23] Gonzalez-Echavarri C Ugarte A Ruiz-Irastorza G. Rituximab-refractory lupus nephritis successfully treated with belimumab. Clin Exp Rheumatol. (2016) 34:355–6. 26886714

[24] Sjöwall C Cöster L. Belimumab may not prevent lupus nephritis in serologically active patients with ongoing non-renal disease activity. Scand J Rheumatol. (2014) 43:428–30. 10.3109/03009742.2014.887769 24689946

[25] Staveri C Karokis D Liossis S-NC. New onset of lupus nephritis in two patients with SLE shortly after initiation of treatment with belimumab. Semin Arthritis Rheum. (2017) 46:788–90. 10.1016/j.semarthrit.2016.09.006 27793432

[26] Kronbichler A Brezina B Gauckler P Quintana LF Jayne DRW. Refractory lupus nephritis: when, why and how to treat. Autoimmun Rev. (2019) 18:510–8. 10.1016/j.autrev.2019.03.004 30844548

[27] GSK Announces Positive Headline Results in Phase 3 Study of Benlysta in Patients With Lupus Nephritis. Available online at: https://www.gsk.com/en-gb/media/press-releases/gsk-announces-positive-headline-results-in-phase-3-study-of-benlysta-in-patients-with-lupus-nephritis/ (accessed March 31, 2020).

[28] Magro-Checa C Zirkzee EJ Huizinga TW Steup-Beekman GM. Management of neuropsychiatric systemic lupus erythematosus: current approaches and future perspectives. Drugs. (2016) 76:459–83. 10.1007/s40265-015-0534-3 26809245 PMC4791452

[29] Farinha F Abrol E Isenberg DA. Biologic therapies in patients with neuropsychiatric systemic lupus erythematosus. Lupus. (2016) 25:1278–9. 10.1177/0961203316631636 26873650

